# Multi Drug and Other Forms of Drug Resistant Tuberculosis Are Uncommon among Treatment Naïve Tuberculosis Patients in Tanzania

**DOI:** 10.1371/journal.pone.0118601

**Published:** 2015-04-07

**Authors:** Tumaini J. Nagu, Said Aboud, Ramadhani Mwiru, Mecky Matee, Wafaie Fawzi, Ferdinand Mugusi

**Affiliations:** 1 Department of Internal Medicine, Muhimbili University of Health and Allied Sciences, Dar es Salaam, Tanzania; 2 Department of Laboratory Medicine, Karolinska Institutet, Stockholm, Sweden; 3 Department of Microbiology and Immunology, Muhimbili University of Health and Allied Sciences, Dar es Salaam, Tanzania; 4 Management and Development for Health, Dar es Salaam, Tanzania; 5 Department of Global Health and Population, Harvard School of Public Health (HSPH), Boston, Massachusetts, United States of America; Indian Institute of science, INDIA

## Abstract

**Background:**

Surveillance and effective management of drug resistance is important to sustaining tuberculosis (TB) control efforts. We aimed to determine resistance rates to first line anti tuberculosis drugs and to describe factors associated with the resistance to any of the first line anti tuberculosis drugs in Dar es Salaam Tanzania.

**Materials:**

Newly diagnosed, TB patients with neither history of tuberculosis treatment nor isoniazid prophylaxis were included into the study. Sputum specimens were cultured on either mycobacteria growth indicator tube 960 (MGIT 960) or Lowenstein Jenstein (LJ) medium supplemented with either glycerol (GLJ) or pyruvate (PLJ). Drug susceptibility for isoniazid, rifampicin, streptomycin and ethambutol was determined by either Lowenstein–Jensen (LJ) medium or mycobacteria growth indicator tube 960 (MGIT 960).

**Results:**

A total of 933 newly diagnosed TB patients, were included into the study. Multi drug resistance (MDR) tuberculosis was detected among 2 (0.2%) patients. Resistance to any of the four tested drugs was detected among 54 (5.8%) patients. Mono-resistance to isoniazid, rifampicin, streptomycin and ethambutol were 21(2.3%), 3 (0.3%), 13 (1.4%), 9 (1.0%) respectively.

**Conclusion:**

Primary resistance to first line anti tuberculosis drugs is still low in this setting. Continued vigilance including periodic national surveillance of anti-tuberculosis resistance is recommended.

## Introduction

Despite near achievement of the 2015 millennia goals in global reduction of tuberculosis (TB) related morbidity and mortality, only 21% of the estimated global cases of multi drug resistance (MDR) TB were identified and treated in 2012.[1] Most of the MDR cases are not detected due lack of surveillance for TB drug resistance in almost half of the World Health Organization (WHO) member states.[2] MDR tuberculosis caused about 170,000 deaths worldwide these statistics suggest that 38% of MDR TB patients died [1] Management of patients with MDR-TB is longer and more complex compared to that of drug sensitive TB.[3]

Recent global antimicrobial reports MDR-TB rate between 3.4–3.6% of new TB patients and 20% of previously treated TB patients had MDR–TB. [2,4] These rates varied from as high as 14.3% among new TB patients in Eastern Europe to as low as 1.9% in African region. [2,4,5] In Sub Saharan Africa (SSA) MDR-TB ranges 1–2% among new treatment and between 6–12% among previously treated TB patients. [6–8] Resistance to any of the first line anti TB treatment has been reported to between 10–27% with isoniazid being the most common in SSA. [5]

Previously conducted studies in Tanzania have reported low rates of drug resistant TB. [9–13] MDR TB was between 0.4–1.6 while INH mono-resistant strains were detected in about 3–4% of newly treated patients. [9–13] Rifampicin mono-resistance seems to be the least common form of drug resistant TB among treatment new TB patients in Tanzania as reported in previous studies. [11,13] Resistance to any of the first line TB drugs among new patients was reported between 5–10%. [9–13].

Increasing urbanization and international migration; coupled with poor infection control could easily spread drug resistant Tuberculosis could globally. Almost all cases of MDR TB in United Kingdom, Belgium and France occurred among immigrants. [14–16] Therefore, need for ongoing monitoring for drug resistance, is indispensable. Nonetheless, continuous surveillance system for anti TB drug resistance is resource intensive, requiring highly qualified manpower and financial ability. This is a challenge in low income countries (LIC). The last TB drug resistance surveillance in Tanzania was conducted 10 years ago. Following this report, there were several initiatives including collaborative TB/HIV activities and introduction of isoniazid prophylaxis therapy in Tanzania. We conducted this study to provide an update on the prevalence of MDR-TB as well as other resistance patterns to first line anti TB drugs among treatment naïve TB patients. We also determined covariates associated with resistance to any of the anti TB drugs. Data generated from this study may serve to inform both national and international policy development. [2]

## Materials and Methods

### Ethics statement

All the research procedures in this study were cleared by the Muhimbili University of Health and Allied sciences (MUHAS) review board prior to commencement of the study (Ref. No. MU/DRP/AEC/Vol.XIII/195). Permission to conduct the study was obtained from the municipal directors as well as clinic management. Informed written consent was obtained from all the patients, in case of patients 15 to 17 years, guardians provided an informed written consent after the minor had accented to participate in the study. Results of MDR were communicated to the attending physicians who followed National guidelines for management of MDR TB. [17]

### Setting and study population

Details of the methods are described in a previously published paper. [18] Briefly, this was a cross-sectional study nested in larger prospective cohort study conducted in 14 urban clinics in Dar es Salaam city, Tanzania between October 2010 and December 2011. According to the National TB records, the selected clinics had highest TB notification rates in the city. Study sites included all three municipal referral hospitals in the city and one clinic responsible for prisons and reprimanded individuals in Dar es Salaam. All study sites are located in urban areas.

Dar es Salaam is the largest commercial city of Tanzania having an important harbor and an international airport. The City has the highest proportion (22%) of all TB cases (61,000) notified in Tanzania in 2011. [19] Therefore, the city is key area for TB control efforts in Tanzania.

To be included, an individual should have been 15 years or older, with at least two positive acid fast bacilli sputum smears on microscopy, attending one of the study clinics and was willing to provide a written consent. We excluded patients who reported history of either previous treatment for TB or isoniazid prophylaxis. All patients were managed according to the national guidelines for TB management. [17] The guidelines demand that patients receive direct observed therapy (DOT) either at health clinic (facility DOT) or supervised by a close relative (community DOT). All patients were treated with isoniazid (H), rifampicin (R), pyrazinamide (Z) and ethambutol (E).

### Sample collection and transportation

Sputum and blood samples were obtained at the TB clinics before treatment commenced. For this study each patient provided one spot sputum sample in addition to routine clinical specimens. Individual specimens were clearly labeled with patients and clinic identification numbers. Samples were received at the laboratory on the same day.

### Sputum Smear

Sputum smears were stained using Ziehl—Neelsen (ZN) stain ZN stain and were examined for acid fast bacilli (AFB) at the TB clinics. Results were reported in the patients’ request forms as well as the laboratory request forms and documented on the clinic TB registers.

### Sputum Culture and drug susceptibility testing

Sputum culture was performed at the Central Reference Tuberculosis Laboratory (CTRL) located at Muhimbili National Hospital. Culture and drug susceptibility test (DST) were performed using either the BACTEC Mycobacteria Growth Indicator Tube (MGIT)—(Beckton-Dickinson) or Lowenstein Jensen (LJ) medium with glycerol (GLJ) and pyruvate (PLJ).

On the LJ media, each sample was cultured at 37°C for up to 8 weeks. Tubes were examined weekly for growth. Colonies were identified based on the speed of growth and macroscopic features e.g. roughness, pigment production and positivity on ZN smear microscopy. Drug susceptibility testing (DST) was done using the proportion method. Standard strain of M tuberculosis H37Rv was tested with each batch of Lowenstein-Jensen Medium. Recommended drug concentrations were used [20]; 0.2mg/l for isoniazid, 40 mg/l for rifampicin, 4 mg/l for streptomycin and 2 mg/l for ethambutol.

MGIT culture was performed using semi-automated MGIT 960 system according to manufacturer instructions. Incubated at 37°C in the semi-automated MGIT 960, positive cultures were inspected for turbidity and cord formation. Mycobacteria culture was confirmed on AFB positivity and negative growth on blood agar.

MGIT 960 system was used to determine DST among 100 patients, LJ method was used for DST among 834 patients while 5 patients had both MGIT and LJ. MGIT and LJ media and the results were (100%) congruent for the 5 patients.

### Other laboratory tests

Hemoglobin was performed using the ACT5 DIFF hematology analyser (Beckman Coulter, Miami, Florida). HIV infection was determined according to the National HIV screening algorithm, serial testing by Determine HIV-1/2 (Inverness Medical Japan Co. Ltd, Japan) then Uni-Gold HIV-1/2 (Trinity Biotech, Wicklow, Ireland). Discordant HIV test results were subjected Enzyme Linked Immunosorbent Assay (ELISA).

### Data processing and analysis

Data were double entered into Epi-info version 6 database by trained data clerks. Every record was uniquely identified using patient identification number. Analysis was performed using SAS version 9.3 (SAS Institute, Carry, NC) statistical software. Proportions for individual drug resistance were calculated. Univariate regression was used to examine the association between ‘any resistance’ (defined as resistance to either H or R or E or S). MDR was defined as resistance to both rifampicin (R) and isoniazid (H). Chi-square (χ^2^) test was used for the comparison of proportions and t-test was used for comparison of means. Log binomial regression was used to examined factors associated with ‘any resistance’. We performed univariate analysis for possible covariates according to literature and multivariate analysis for covariates associated with ‘any resistance’ with p< 0.3 at univariate analysis. Two tailed P < 0.05 was considered significant.

## Results

During the study period, October 2010—December 2011, a total of 1805 patients were recruited for the prospective TB study. Of these, 1668 (92%) patients supplied sputum for culture. Mycobacteria tuberculosis (MTB) was isolated from 1365 (82%) patients, while 244 (15%) patients were negative for MTB, four patients had mycobacteria other than tuberculosis (MOTT) and 55 (3%) cultures were contaminated. Among the MTB culture positive patients, 939 (69%), were subjected to drug susceptibility testing (DST) with first line anti tuberculosis drugs, isoniazid (H), rifampicin (R), streptomycin (S) and ethambutol (E). Six samples were irreversibly contaminated at DST, we report results from 933 patients.

In this study population the mean age was 33 ±11 years and about two thirds of the patients 628 (68%) were male. HIV positive TB patients comprised one third 270 (30%) out of whom only 24 (9%) were registered in HIV treatment clinics and had started using anti-retroviral treatment (ART) at the initiation of their TB treatment. Generally the study population had a relatively poor nutritional status; mean hemoglobin 10.4g/dl and mean body mass index (BMI) 18.9kg/m^2^. More than 70% had tuberculosis symptoms for four weeks or more. The study group to a large extent represent the lower socioeconomic class, 704 (78%), had primary education or below with a median family income of 100 USD per month and median family daily expenditure on food about 3 USD. Details of characteristics of the study group are provided in Table 1.

**Table 1:** Basic characteristics of adults patients with pulmonary tuberculosis in Dar es Salaam, Tanzania (n = 933).

Characteristics	Missing n (%)	n (%) / mean (SD)
Mean age in years (SD)	27 (2.9)	33.6 (11.4)
Age groups (years)	27 (2.9)	
15 - <30		373 (41.7)
30–50		463 (51.1)
>50		70 (7.7)
Male sex n (%)	0	632 (67.7)
Education n (%)	23 (2.5)	
No formal education		50 (5.5)
Primary school		658 (72.3)
Secondary and above		202 (22.2)
Marital status (%)	17 (1.8)	
Never married		426 (46.5)
Married/cohabiting		401 (43.8)
Divorced/widowed		89 (9.7)
Smoking status n (%)	13 (1.4)	
Never		667 (72.5)
Past		201 (21.9)
Current		52 (5.7)
Alcohol drinking status n (%)	20 (2.1)	
Never		590 (64.6)
Past		256 (28.0)
Current		65 (7.3)
HIV positive n (%)	50 (5.4)	270 (30.7)
Mean BMI (kg/m^2^)	125 (13.4)	18. 9 (3.7)
BMI groups	125 (13.4)	
<18.5		422 (52.2)
18.5–24.99		349 (43.2)
≥25		37 (4.6)
Mean Hemoglobin (g/dl)	133 (14.3)	10.4 (2.2)
Anemia	133 (14.3)	
Yes		688 (86.0)
No		112 (14.0)
Median family income per month (USD)	206 (22.1)	100
Average family income per month (USD)	206 (22.1)	
< 100		325 (44.7)
≥ 100		402 (55.3)
Median daily expenditure on food (USD)	146 (15.6)	3
Average daily expenditure on food (USD)	146 (15.6)	
< 3		334 (42.4)
≥ 3		453 (57.6)
Duration of illness (weeks)	104 (11.1)	
< 4		209 (25.2)
≥ 4		602 (74.8)

Table 2 displays resistance patterns to H, R, E and S. Resistance to at least one drug was detected in 54 (5.8%) patients. Any and mono resistance to isoniazid was found in 28 (3.0%) and 21 (2.3%) respectively. Rifampicin resistance was detected in 5 (0.5%) patients out of whom two (0.2%) were multi drug resistant (MDR) TB. All the patients with MDR-TB had concurrent streptomycin resistance.

**Table 2:** Patterns of primary resistance to first line anti tuberculosis drugs among adult patients with pulmonary tuberculosis in Dar es Salaam, Tanzania (n = 933).

	N	%
Total number isolates	933	100
**Any resistance**	**54**	**5.8**
H	28	3.0
R	5	0.5
S	18	1.9
E	12	1.3
RH	31	3.3
**Mono resistance**	**46**	**4.9**
H	21	2.3
R	3	0.3
S	13	1.4
E	9	1.0
**Multidrug resistance**	**2**	**0.2**
HR	0	0.0
HRS	2	0.2
HRSE	0	0.0
**Poly resistance**	**5**	**0.5**
HE	3	0.3
HS	2	0.2

Table 3 shows univariate analysis of baseline covariates associated with any resistance to first line anti TB drugs tested. None of the covariates was statistically associates with any resistance to H,R,E and S.

**Table 3:** Univariate and multivariate analysis for factors associated with resistance to first line anti-tuberculosis drugs in Dar es salaam, Tanzania (n = 933).

Variable	Univariate		Multivariate	
	RR (95% CI)	P	RR (95% CI)	P
Age (years)	0.99 (0.97–1.01)	0.78		
Age groups (years)		0.60		
15 - <30	1			
30–50	0.94 (0.55–1.62)			
> 50	0.69 (0.21–2.25)			
Sex		0.86		
Male	0.95 (0.53–1.70)			
Female	1			
Marital status		0.41		
Never married	1			
Married /Cohabiting	1.32(0.75–2.35)			
Divorced/widowed	1.68 (0.73–3.84)			
Education achieved		0.71		
None	0.35(0.05–2.59)			
Primary	1.05 (0.57–1.93)			
Secondary & Tertiary	1			
Smoking status		0.85		
Never smoker	1			
Past smoker	1.25 (0.68–2.25)			
Current smoker	0.70 (0.17–2.77)			
Alcohol drinking status		0.68		
Never drinker	1			
Past drinker	1.36 (0.75–2.25)			
Current drinker	0.83 (0.26–2.62)			
HIV status		0.55		
Positive	0.80 (0.44–1.55)			
Negative	1			
Duration of illness (weeks)		0.51		
< 4	1			
≥ 4	1.27 (0.49–1.50)			
Mean BMI (kg/m^2^)	1.03 (0.97–1.09)	0.37		
BMI groups (kg/m^2^)		0.06		0.24
<18.5	0.43 (0.14–1.33)		0.99 (0.56–1.84)	
18.5–24.99	0.66 (0.22–2.01)		1.94 (0.61–6.16)	
≥25	1		1	
Mean Hemoglobin (g/dl)	0.92(0.81–1.05)	0.25	0.92 (0.81–1.06)	0.25
Anemia status		0.96		
Yes	0.98 (0.40–2.37)			
No	1			
Average family income per month (USD)		0.59		
< 100	0.86 (0.49–1.50)			
≥ 100	1			
Average daily expenditure on food (USD)		0.17		0.76
< 3	1.43 (0.85–2.41)		1.09 (0.59–2.01)	
≥ 3	1		1	

## Discussion

We describe primary resistance to isoniazid, rifampicin streptomycin and ethambutol in TB population where a third of patients are co-infected with HIV. There three important findings worth noting from this study. First, MDR and other forms of resistance are low in treatment naïve TB patients in this setting. Secondly, we did not find any factor associated with occurrence of drug resistant (HRES) TB in this population. Third, we take note that TB clinics continue to serve as important entry point to HIV care and treatment.

Our finding of low MDR–TB in this setting are similar to previously published work by Urassa et al [9] who documented 0.4% of new TB patients in Dar es Salaam had MDR–TB. [9] MDR–TB rates we report are slightly lower than previous reported rates between 1.2–1.6% among new TB patients in Tanzania. [11,13] The highest MDR–TB in Tanzania was reported by the national surveillance study. [13] By and large, all these studies point out to lower MDR among new treatment TB patients. Neighboring countries of Kenya, Uganda and Malawi MDR–TB in Tanzania reports similar low rates of MDR between 0.6–1.4% among new TB patients. [7,8]

Resistance to any of first line anti TB drugs was (R, H, S, E) similarly low, comparing well with Urassa et al [9] and lower than other local studies [11, 13] at the similar drug concentration to our current study. Similar to previous national and international studies, isoniazid resistance was the most frequent observation in this study population. Since isoniazid is used for prophylaxis among contacts of open TB patients and is now increasingly used for latent TB infection among HIV infected individuals, this fact should raise an alarm. [21] Our study excluded patients with any previous TB treatment as well as self-reported isoniazid prophylaxis. These stringent inclusion criteria may therefore have contributed to slightly lower resistance rates we report in the current study. Earlier studies in Tanzania [11–13] did not categorically distinguish treatment naïve from other patients previously treated and this may contribute to misclassification in the previous studies.

The rates of drug resistant TB has remained low in Tanzania for a number of years. [9–13] This could be a result of tight TB drug management protocols and DOT spear headed by the National TB Programme. We can’t however, rule out the contribution of molecular characteristics of the circulating mycobacteria strains and that of the host to explain the low rates of drug resistant TB in this setting. Genetic studies would be important in this facet.

We did not find any factors associated with primary anti-TB resistance among our study group. Relapse and previous treatment with TB drugs have been shown to be most important factors associated with TB drug resistance. [7,8,22] We excluded previous treatment and that may partly explain the lack of associations. The absence of associated covariates which could alert for further resistance testing, underscores the importance of having drug resistance surveillance mechanism in place.

This study has demonstrated that, ten years following widely scale up of anti-retro viral therapy (ART) in Tanzania, TB clinics are important entry points for HIV treatment. Less than one in ten of HIV patients in our study population were registered with HIV clinics and were on ART. This means that majority of the HIV patients could be linked to HIV care through TB services if national policy for continuum of care is implemented. [21] Emphasis should aim to link and retain these patients to HIV services as a strategy to reduce both HIV and TB transmission.

Our study has several strengths. First, it is yet another effort in the surveillance of drug resistance to TB, a call that echoes worldwide. [2] Second, inclusion of all the three Municipal referral hospitals and representation of all the three municipals by primary health facilities envisage that the findings can easily be generalized in the whole of Dar es Salaam City. Since Dar es Salaam represents about one fifth of all TB cases in Tanzania in a year, our findings can be used to estimate the burden of MDR in other parts of the country. Third, a large sample size suggests that our estimates are likely to be closer to the true estimates in the study settings. Fourth, this study provides detailed description of the study population including socio-demographic and nutritional characteristics which missed in almost all the previous studies in Tanzania.[11–13] Description of the study population gives a better insight of the resistance rates obtained, makes it easier for comparison with other studies and accessible for review studies and meta-analysis as need arise. Fifth, we also attempted to find predictors for the occurrence of anti-TB resistance. The major weakness of this study is exclusion of almost 50% of the recruited patients (n = 1805) from DST studies. The patients included and those excluded from this study did not differ by their mean age, sex, HIV status, BMI, hemoglobin and average daily expenditure on food. It is therefore unlikely exclusion would alter the prevalence of anti-TB resistance.

## Conclusion

MDR and other forms of first line drug resistant TB infrequent among treatment naïve TB patients in this population. Emphasis should be placed on maintaining these rates including continued surveillance programs. Molecular studies are recommended to describe determinants of drug resistant Tuberculosis and define novel mechanisms of resistance and virulence factors.

## Supporting Information

S1 Dataset Dataset for the study. (SAS7BDAT)
